# On-farm biosecurity as perceived by professionals visiting Swedish farms

**DOI:** 10.1186/1751-0147-56-28

**Published:** 2014-05-09

**Authors:** Maria Nöremark, Susanna Sternberg-Lewerin

**Affiliations:** 1 Department of Disease Control and Epidemiology, SVA, National Veterinary Institute, Uppsala SE-751 89, Sweden; 2 Department of Biomedical Sciences and Veterinary Public Health, SLU, Swedish University of Agricultural Sciences, Box 7028, Uppsala SE-750 07, Sweden

## Abstract

**Background:**

On-farm biosecurity is an important part of disease prevention and control, this applies to live animal contacts as well as indirect contacts e.g. via professionals visiting farms in their work. The objectives of this study were to investigate how professionals visiting animal farms in Sweden in their daily work perceive the on-farm conditions for biosecurity, the factors that influence their own biosecurity routines and what they describe as obstacles for biosecurity. Suggestions for improvements were also asked for. Questionnaires were distributed to professionals visiting farms in their daily work; veterinarians, livestock hauliers, artificial insemination technicians, animal welfare inspectors and cattle hoof trimmers. The sample was a convenience sample, based on accessibility to registers or collaboration with organisations distributing the questionnaire. Respondents were asked about the availability of certain biosecurity conditions related to farm visits, e.g. if facilities for hand washing were available, how important different factors were for their own routines and, through open ended questions, to describe obstacles and suggestions for improvement.

**Results:**

After data cleaning, there were responses from 368 persons. There was a difference in the proportion of visited farms reported to have certain biosecurity measures in place related to animal species present on the farm. In general, visited pig farms had a higher proportion of biosecurity measures in place, whereas the conditions were poorer on sheep and goat farms and horse farms. There were also differences between the visitor categories; the perceived conditions for biosecurity varied between the groups, e.g. livestock hauliers did not have access to hand washing facilities as often as veterinarians did. In all groups, a majority of the respondents perceived obstacles for on-farm biosecurity, among veterinarians 66% perceived that there were obstacles. Many of the reported obstacles related to the very basics of biosecurity, such as access to soap and water. Responsibility was identified to be a key issue; while some farmers expect visitors to take responsibility for keeping up biosecurity they do not provide the adequate on-farm conditions.

**Conclusions:**

Many of the respondents reported obstacles for keeping good biosecurity related to on-farm conditions. There was a gap when it came to responsibility which needs to be clarified. Visitors need to take responsibility for avoiding spread of disease, while farmers need to assume responsibility for providing adequate conditions for on-farm biosecurity.

## Background

Indirect contacts via visitors can play a role in the spread of both endemic and exotic diseases. Adequate biosecurity routines can minimize the risk of such spread, e.g. using clean boots and protective clothing and cleaning equipment between farms. Correspondingly, a lack of biosecurity can contribute to the spread of disease [1-6].

The importance of farm biosecurity has been highlighted during the last decade; within the European Union (EU) the proposal for a new animal health law emphasises biosecurity [7] and a number of studies with focus on-farm biosecurity have been published [3,8-20]. Specifically related to farm visits and biosecurity for visitors, Racicot and co-authors have in a series of studies analyzed biosecurity errors while entering farms and factors affecting compliance with routines, e.g. personality traits and education [21-23].

In a previous study conducted in Sweden [24], disparities were found in biosecurity routines between farmers with different species and different herd sizes. The farmers also reported a difference in the biosecurity routines applied by different categories of professional visitors coming to their farms, for example livestock hauliers, veterinarians and inspectors. Some farmers reported that they had different requirements for biosecurity depending on type of visitor. Moreover, some farmers reported that they did not consider biosecurity necessary unless there were current outbreaks of exotic diseases in the country. When working with outbreak investigations, the authors have experienced that professional visitors sometimes adapt their routines depending on the farmers’ requirements, the same person can thus have very different routines in different farms. This interaction between the farmer and the visitor is part of the complexity related to the on-farm biosecurity applied by visitors. Several different factors could influence the intended behaviour of the visitor, such as the requirements from their own organization and their own will not to spread disease. But regardless of the visitors’ own intentions, the practical and physical conditions provided on the farms, as well as requirements, or lack thereof, from the farmers, will probably affect what is actually done on each farm. Practical obstacles can impair the intended behaviour. If the visitor does not have access to running water while on the farm, washing the boots before leaving will be difficult. In a Canadian study it was shown that design of the hygiene barrier affected the number of biosecurity errors made by visitors [22].

Although many diseases are species specific, this does not apply to all diseases. Some visitors, e.g. veterinarians, often visit many different categories of farms and could potentially spread disease between different species or different categories of the population. If the routines for biosecurity are inferior in one type of species, this could thus impact spread of disease to other species. Some of the diseases are also zoonotic; several studies have identified a higher prevalence of zoonotic diseases among veterinarians, and concern among veterinarians for contracting zoonotic infections has also been investigated [25-28].

In Sweden, as well as in other parts of the EU, several projects are currently underway to improve on-farm biosecurity routines, decrease the risk of spread of endemic livestock diseases and decrease the risk of outbreaks of exotic diseases. The proposal for a new EU Animal Health Law puts more responsibility for disease prevention on the farmers and, consequently, a high level of on-farm biosecurity will be required when the regulation comes into force [7].

The objectives of this study were to investigate how professionals visiting farms with animals in Sweden in their daily work perceive the on-farm conditions for biosecurity, the factors that influence their own biosecurity routines and what they describe as obstacles for biosecurity, and to collect suggestions for improvements. The aim is to use the information as a basis for future work in improving on-farm biosecurity and biosecurity among professional visitors.

## Methods

### Sample and distribution of questionnaire

Data in this study were gathered through questionnaires sent to five categories of professionals that regularly visit farms in their work. These were; veterinarians, livestock hauliers, artificial insemination (AI) technicians, animal welfare inspectors and cattle hoof trimmers. This sample was a convenience sample of groups where some kind of contact information or distribution channel was found. The chosen categories were either included in an accessible official register (livestock hauliers), or were part of organizations that were willing to distribute the questionnaire among their employees or members (veterinarians, AI-technicians, animal welfare inspectors), or had contact information available on websites (hoof trimmers). For this reason the exact number of persons receiving the questionnaires within each category is not known. For veterinarians, e-mail questionnaires were distributed by the two Swedish veterinary unions (without indicating the exact number of recipients, one of the unions reported they sent to all members and the other one to members of the sections for livestock and horse practitioners). Livestock hauliers were identified through an official register held by the Swedish Board of Agriculture and were sent the questionnaire either via e-mail (n = 104), in paper format (n = 40), or both paper and e-mail (n = 35) depending on the data received from the register; not all livestock hauliers had e-mail addresses. Three AI companies, one national and two regional, distributed the electronic version of the questionnaire among their employees (the exact number of recipients is not known). Animal welfare inspectors in Sweden work in the County Administrative Boards, and were contacted through the network of the heads of animal welfare in these authorities. The questionnaire was distributed within their network and redistributed at regional level by their contact points (exact number of recipients is therefore not known). Cattle hoof trimmers were identified through a cattle hoof trimmers organization webpage and, after a search of addresses using an internet search engine, questionnaires were sent to all e-mail addresses obtained (n = 57). The questionnaires were distributed between May and December 2012. An invitation letter was attached to the questionnaire which explained the background of the study, clarified that answers would be treated anonymously and encouraged participation. Reminders were sent to hoof trimmers and some of the livestock hauliers for which there was direct access to the e-mail addresses. Since other groups received the questionnaire through their organisations, sometimes forwarded in several steps, reminders were not sent to these groups.

The first section of questions related to the on-farm conditions. Respondents were asked to give the proportions of farms they visit that have; hygiene barrier, protective clothing for visitors, hand-washing facilities, hand disinfection, and requirements as regards to the use of protective clothing. These questions were split by species present on the farms (cattle, pigs, sheep/goats, horses) and respondents were asked to respond only for the farms that they normally visit in their daily work. Livestock hauliers were also asked how often they needed to enter animal buildings. The second part of the questions related to the visitors’ own routines; how important different factors were for their routines, and the proportion of visits when they applied different routines. The third, and last, part consisted of open ended questions. Respondents were asked if there were any diseases that they in their profession were afraid to spread between farms or contract themselves. Finally they were asked about obstacles for biosecurity and factors for improving biosecurity, both on farm level and within their own profession. The questionnaire also included background questions on profession, age, number of farms visited per week. The questionnaire is available as Additional file 1 (in English) and Additional file 2 (in Swedish). Before sending the questionnaire, a pilot version was tested on 14 veterinarians working in the National Veterinary Institute. The group was a mixture of persons with recent experience of working in the field and visiting farms on a daily basis, with expertise in biosecurity or with experience in questionnaire design. Data from the paper questionnaires were entered manually into the electronic version of the questionnaire, data entry was checked for consistency.

### Analysis

The data were checked and cleaned; data from respondents that were not part of the intended study population were dropped. Descriptive statistics were obtained for all closed questions, both total and by categories of visitors. The responses to all open questions were read separately by the two authors, who created different categories representing the different types of responses. These categories were thereafter compared and merged into a single list by the two researchers. Each response was then assigned into one or more of the categories, this was also done separately by the two authors and the results were then checked for consistency. Whenever there was a discrepancy, this was discussed and the response was assigned to one of the categories. Finally, the frequency of the different response categories was calculated. Some replies were combined, for example, ‘MRSA’ (Methicillin resistant *Staphylococcus aureus*) were combined with ‘multiresistant bacteria’, thus anyone replying both ‘MRSA’ and ‘multiresistant bacteria’ contributed only once to ‘multiresistant bacteria or MRSA’.

### Software used

The questionnaire was designed and administered using the web survey software Easyresearch (QuestBack International HQ, Oslo, Norway). Data were analysed using STATA 11.2 (Stata Co., College Station, Texas, USA), graphs were drawn using Microsoft Office Excel 2007 (Microsoft Co., Redmond, USA).

## Results

### Response rate

The numbers of respondents (to the entire or part of the questionnaire) after data cleaning were; 188 veterinarians, 82 animal welfare inspectors, 59 AI-technicians, 28 livestock hauliers and 11 cattle hoof trimmers, in total 368. Seven respondents were dropped during data cleaning because they either did not visit farms, worked abroad or belonged to a profession that was not included in the sample. The age of the respondents was as follows; 20% were 20–35 years, 35% 36–50, 41% 51–65 and 4% >66 years. The age varied between the groups; 63% of the AI-technicians, 52% of the hauliers and 51% of veterinarians were above 50 years of age, while only 36% of the hoof trimmers and 17% of the inspectors were above 50 years of age. Since the sample was a convenience sample and data collection relied on the collaboration with organizations and their willingness to distribute the questionnaire, the number of persons that received the questionnaire within each category for veterinarians, AI-technicians and animal welfare inspectors was not known. For the categories with direct access to e-mail addresses; cattle hoof trimmers and some of the livestock hauliers, the response-rate was low and some e-mails also bounced, indicating the addresses were no longer in use. For hoof trimmers 27% of the addresses bounced and based on the addresses that did not bounce, the response rate was 17%. For the livestock hauliers that only received the questionnaire through e-mail, 21% of the addresses bounced and the response rate was 5%. However, for the paper questionnaires to livestock hauliers the response rate was 27%.

### Descriptive statistics

Cattle farms were visited by most respondents (86%), while pig farms were visited by the least (43%). The number of farms visited varied, with hauliers and AI-technicians visiting most farms; 56% and 69% of them visited more than 20 farms per week (Table 1).

**Table 1:** Types of farms visited and number of farms visited per week by professionals responding to the questionnaire

	Proportion* of respondents reporting to visit each type of farm (%)				**Number of farms reported to be visited per week (%)**				
**Category of visitor**	**Cattle**	**Pig**	**Sheep/goat**	**Horse**	**<1**	**1-10**	**11-20**	**>20**	**n**
Veterinarian	77	33	57	87	12	46	34	9	181
AI-technician	100	15	0	3	2	5	24	69	59
Animal transporter	82	71	68	21	0	11	33	56	27
Inspector	96	79	96	95	10	89	0	1	81
Cattle hoof trimmer**	90	27	9	9	9	81	9	0	11
Total	86	43	56	68	9	47	24	20	359

*One or more type could be indicated by the respondent. **One cattle hoof trimmer worked with species other than cattle.

**Figure 1:**
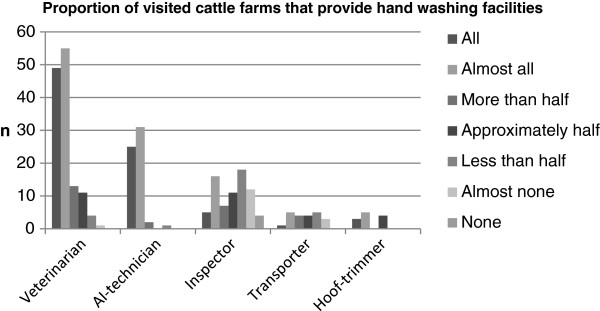
Proportion of visited cattle farms that provide hand washing facilities, according to different groups of professionals visiting farms in their work.

The reported on-farm biosecurity differed depending on species present on the farm (Table 2). In general, the highest proportion of biosecurity measures related to farm visits were reported to be in place on pig farms, followed by cattle farms. Farms with small ruminants or horses were reported to have less biosecurity measures in place. On these farms it was reported that there was seldom access to protective clothing or boots for the visitors, or even possibility for hand washing. Among veterinarians, a group in close contact with sick animals, 24% (n = 152) reported that hand washing facilities were available on none or almost none of the horse farms they visit. The corresponding figure for sheep farms was 31% (n = 100). There were also reported differences within the same farm types, i.e. different categories of visitors reported different proportions of certain biosecurity measures to be present on the farms they visit. This is illustrated through one example in Figure 1, showing availability of hand washing facilities on cattle farms. A clear majority of veterinarians and AI-technicians reported this to be available on all or almost all cattle farms they visit, which was in clear contrast to inspectors and livestock hauliers. A similar picture was also seen in pig farms. Among the veterinarians, 49% (n = 57) reported they could wash their hands on all pig farms they visit, but only 6% (n = 17) of the hauliers. In fact, 35% of the livestock hauliers reported they could wash their hands on none or almost none of the pig farms they visited. A clear difference was also seen for availability of protective clothing in cattle herds, where 81% (n = 21) of the hauliers reported protective clothing to be available on none or almost none of the cattle farms they visited, but only 21% (n = 57) of the AI-technicians reported it to be available on almost none of the farms (0% answered ‘none’). Corresponding patterns for protective clothing were seen on pig farms when comparing livestock hauliers and veterinarians; 41% of hauliers (n = 17) and 11% of veterinarians (n = 56) reported protective clothing to be available on none or almost none of the farms. Similar patterns were seen when comparing reported availability of boots for visitors both on cattle and pig-farms. On cattle farms 76% (n = 21) of the hauliers, 44% (n = 133) of the veterinarians and 37% (n = 59) of the AI-technicians reported that boots were available on none or almost none of the farms. For pig farms 63% (n = 57) of the veterinarians and 6% of the hauliers (n = 18) reported boots to be available on all or almost all farms.

**Table 2:** Proportion of Swedish farms visited by professionals in their work reported to have certain biosecurity measures in place

	**% responses in each category**							
**Question**	**All**	**Almost all**	**More than half**	**Approximately half**	**Less than half**	**Almost none**	**None**	**n**
**When visiting farms, proportion of farms that have:**								
**Hygiene barrier**								
Pig farms	15.4	33.1	13.2	10.3	9.6	14.0	4.4	136
Cattle farms	0.7	7.2	3.2	3.2	18.7	44.6	22.3	278
Sheep/goat farms	0	1.1	0	0	7.3	37.2	54.5	191
Horse farms	0	0	0.4	0	1.3	14.8	83.5	230
**Protective clothing for visitors**								
Pig farms	18.0	25.0	16.4	5.5	11.7	14.8	8.6	128
Cattle farms	0.4	11.0	4.2	6.0	26.2	35.3	17.0	283
Sheep/goat farms	0	0	0.5	0	4.3	30.8	64.3	185
Horse farms	0	0	0	0	0	9.0	91.0	234
**Boots for visitors**								
Pig farms	18.6	24.8	14.0	6.2	10.1	17.8	8.5	129
Cattle farms	0.4	1.7	4.8	6.6	30.5	39.8	16.3	289
Sheep/goat farms	0	0	0	0	4.3	28.1	67.6	185
Horse farms	0	0	0	0	0.0	7.4	92.6	231
**Facilities for hand washing**								
Pig farms	28.4	33.6	13.4	3.0	11.2	7.5	3.0	134
Cattle farms	28.0	37.8	7.4	9.1	9.1	6.1	2.4	296
Sheep/goat farms	1.6	10.2	6.4	10.7	21.9	34.2	15.0	187
Horse farms	1.7	8.2	6.5	22.1	29.9	19.9	11.7	231
**Hand disinfection**								
Pig farms	4.4	10.6	7.1	5.3	13.3	36.3	23.0	113
Cattle farms	1.1	1.1	3.2	5.7	15.0	48.2	25.7	280
Sheep/goat farms	0	0.6	0.6	1.7	4.6	25.6	67.1	176
Horse farms	0	0.5	0	2.3	5.4	28.4	63.5	222
**Proportion of the farmers who require that visitors use protective clothing:**								
Pig farms	27.9	32.6	7.8	12.4	6.2	8.5	4.7	129
Cattle farms	8.4	20.9	10.6	7.0	16.5	28.9	7.7	273
Sheep/goat farms	0.6	2.9	4.6	9.2	12.6	35.6	34.5	174
Horse farms	0.4	0	0	0.9	2.6	29.2	67.0	233

Based on a questionnaire to veterinarians, AI-technicians, animal welfare inspectors, livestock hauliers and hoof trimmers in 2012–2013.

When it came to biosecurity requirements made by farmers, there was also a perceived difference between the groups of visitors with 73% (n = 22) of hauliers reporting that none or almost none of cattle farmers they visit have any biosecurity requirements, whereas the other professionals reported a higher proportion of cattle farmers requiring biosecurity measures. Requirements also differed between animal species on the farms. Hauliers reported 38% (n = 16) of pig farmers to have biosecurity requirements. Of the veterinarians, 75% (n = 152) reported that none of the horse farms they visit have biosecurity requirements.

As for entering the farm buildings (a question that was specifically asked to livestock hauliers since they sometimes enter farms), there was a difference between pig farms compared to cattle, sheep and goat farms. Only 4% reported that they entered almost all pig farms (no-one wrote they entered all pig farms) whereas 43% wrote they entered all or almost all cattle farms and 39% that they entered all or almost all sheep and goat farms (n = 28).

For the factors affecting their own biosecurity routines, all categories of visitors had a high proportion of respondents reporting that ‘their own will not to spread disease’ (82-96%, total 95% n = 345) and ‘current disease outbreaks’ (91-95%, total 93% n = 341) were very important for their routines. Animal species present on the farm and herd size were regarded as very important by 28% (n = 336) and 15% (n = 343) of the respondents respectively. Again, there was a difference between groups. Only 4% (n = 28) of the livestock hauliers reported ‘farmers’ requests on biosecurity’ to be ‘irrelevant’ or ‘less important’ for their routines, whereas the corresponding figure for the inspectors was 35% (n = 77). Among both the AI-technicians (n = 57) and the hoof-trimmers (n = 11) 91% reported that the “market advantage of keeping a good hygiene” was ‘quite important’ or ‘very important’ for their routines, compared to 59% (n = 170) of the veterinarians.

There was also a difference between groups whether they had asked the farmer to improve the routines or not, with veterinarians being the group most often (56%, n = 186) reporting that they had asked farmers to improve the farm conditions for biosecurity for visitors ‘many times’. Only 11% (n = 28) of the livestock hauliers had asked this ’many times’. Among the inspectors, 60% (n = 82) had never asked farmers to improve the on-farm conditions for visitors’ biosecurity.

When assessing their own routines, 18% considered them to be ‘very good’, 58% ‘sufficient’, 8% ‘insufficient’ and 16% said this varied between farms (n = 343).

When looking at the perceived risk of spreading or contracting disease, there were also differences between the groups. In all groups, a majority (by group 54-87%; total 76%, n = 338) reported that there were infectious agents that they were afraid to spread between farms, with the highest number among veterinarians (87%, n = 146). For all the groups, there were higher proportions reporting they were afraid to spread disease, as compared to contracting disease themselves (21-63%, total 44%, n = 340). Among the respondents being afraid to spread or contract diseases, 205 gave examples of what they were afraid to spread (Table 3), and 128 gave examples of what they were afraid to contract (Table 4). The infections that the visitors were most afraid to spread between farms were; salmonellosis and ringworm followed by strangles and less well specified infections such as ‘diarrhoea’, ‘viral diseases’, and ‘respiratory diseases’. The same two infections, but in reversed order were in the two top positions for what the visitors were afraid to contract; ringworm first and in second place salmonellosis. These were followed by multiresistant bacteria and MRSA, EHEC, and listeriosis.

**Table 3:** Infectious agents or diseases professionals visiting farms in their work reported to be afraid to spread

**Infectious agents or disease-conditions professionals visiting farms in their work reported to be afraid to spread**	**Number of times stated**
*Salmonella*	72
Ringworm	59
Diarrhoeal diseases	57
Strangles	50
Viral diseases	40
Respiratory diseases	35
Respiratory syncytial virus	22
Everything	20
Corona virus	19
Equine influenza	15
MRSA, multiresistant bacteria, ESBL	15
BVDV	14
Equine herpes virus	13
Influenza	11
VTEC/EHEC	8
Ongoing infections	7
Epizootic diseases	6
Equine viral diseases	6
Foot rot	5
Unknown diseases	5
Other*	62

Based on an open ended question in a questionnaire to Swedish veterinarians, AI-technicians, animal welfare inspectors, livestock hauliers and hoof trimmers in 2012–2013. Responders were asked to give examples (without ranking) of diseases they were afraid to spread in their work.

In total 205 responders gave one or more examples of what they were afraid to spread (121 veterinarians, 38 AI-technicians, 26 inspectors, 11 hauliers, 9 hoof trimmers) *Other = disease or disease condition reported by less than five responders, e.g.: Bacterial diseases; Mastitis; Swine dysentery; Digital dermatitis; Maedi Visna; PRRS; APP; *Fusobacterium necroforum*; Flees; Papilloma virus; Q-fever; Swine influenza; BSE; CEM; Circo virus; Malignant catarrhal fever; *Lawsonia intracellularis*; Bovine leukosis; Foot- and mouth disease; Rhinovirus; Rota virus; *Erysepelotrix rhusopatie*; SEP, Scabies, Streptococci.

**Table 4:** Infectious agents or disease-conditions professionals visiting farms in their work reported to be afraid to contract

**Infectious agent, disease or condition responders were afraid to contract**	**Number of times stated**
Ringworm	55
*Salmonella*	39
MRSA, Multi-resistant bacteria and ESBL	22
EHEC	21
Listeriosis	10
Q-fever	6
Rabies	6
Wound infections	5
Toxoplasmosis	5
Diseases that are a risk when pregnant	5
Other*	41

Based on an open ended question in a questionnaire to Swedish veterinarians, AI-technicians, animal welfare inspectors, livestock hauliers and hoof trimmers in 2012–2013. Responders were asked to give examples (without ranking) of diseases they were afraid to contract in their work.

In total, 128 responders (73 veterinarians, 25 AI-technicians, 18 inspectors, 7 hoof-trimmers, 5 hauliers) gave one or more examples *Other = disease or disease condition reported by less than five responders, e.g.: Bacteria; Tuberculosis; Anthrax; *Campylobacter*; Cryptosporidium; Orf; Mycoplasma; Scabies, Intestinal bacteria; *Toxocara cati*; Diseases related to abortion.

In total approximately half (52%, n = 338) of the respondents reported that there were obstacles to keeping a high level of biosecurity during farm visits. The number of respondents reporting obstacles was clearly highest among the veterinarians; 66% (n = 169) compared to 33-40% among the other groups. Of the respondents, 178 gave examples of obstacles and the majority of the examples (213) were related to conditions on the farm (Table 5). With the number one being ‘Lack of water, soap, wash basin, paper towels’, followed by ‘Inadequate equipment or lack of water to clean boots or equipment’ and in third place ‘Adequate protective clothing not available on the farm; non-existing, cold, dirty or wrong size’. There were also obstacles related to the working situation (22); ‘Lack of time, the working schedule does not allow adequate cleaning between farms’ or ‘Inadequate protective clothes, or not as many as needed provided by the employer’. Also for the suggestions on improvement (Table 6), measures related to conditions on the farm dominated (235) followed by measures related to communication (84). For the farm related suggestions, most related to ‘Protective clothing made available on-farm; clean, warm and of adequate size’ (65), followed by ‘Warm water, soap, wash basin and paper-towels available on-farm’ (57) and ‘Hard surface and water hose with adequate pressure available on the farm to clean boots and equipment, adequately located’ (34). Regarding communications there were suggestions on ‘Information to farmers, making farmers more aware, active dialogue with farmers, move the responsibility to the farmers’ (58) as well as ‘National biosecurity guidelines for both farmers and professionals’ (9).

**Table 5:** Reported obstacles for on-farm biosecurity reported by professionals visiting farms in their work

**Reported obstacles for on-farm biosecurity**	**(n)**
Lack of water, soap, wash basin, paper-towels (f)	81
Inadequate equipment or lack of water to clean boots or equipment (f)	51
Adequate protective clothing not available on the farm; non-existing, cold, dirty or wrong size (f)	30
Lack of time, the working schedule does not allow adequate cleaning between farms	18
No hygiene barrier or inadequate separation of clean and dirty areas (e.g. have to pass dirty area after washing) (f)	17
Sensitive equipment (e.g. handheld computer) is difficult to clean	15
Ignorance, unwillingness or unawareness among the farmers	14
No clean surfaces for equipment, e.g. no clean table for the veterinary medical equipment (f)	13
Cold climate makes it difficult to change on-farm, cause chapped hands, shoe covers are slippery on snow and ice, water freezes when washing equipment	12
No hand disinfectant available on-farm (f)	7
Lack of space in the car (w)	7
Inadequate protective clothes, or not as many as needed provided by the employer (w)	6
Lack of general hygiene on-farm (f)	5
Other*	40

Based on an open ended question in a questionnaire to Swedish veterinarians, AI-technicians, animal welfare inspectors, livestock hauliers and hoof trimmers in 2012–2013.

(f) = farm related, (w) = workplace related.

*Other = Obstacles reported by less than five responders, e.g.: Dirty yard; If demands are too high, one risks to be regarded as uncomfortable and the farmer will turn to someone else instead; Standard protective coat scares some horses; No separate out load area for dispatched animals; The farmers are afraid of the animals and cannot load them; Distance prevents planning of trips based on biosecurity levels; Design of the building; The farmer does not inform about current disease situation on the farm; Lack of resources (money); Shared vehicles; Disposable shoe protections of poor quality; Lack of guidelines on national level for farmers and professionals visiting farms in their work/the requirements are too low; Lack of hygiene education; Inadequate handling of laundry, protective clothing are re-contaminated; Difficult to find adequate protective clothing for cold winter climate; Animal welfare inspection visits often have a negative effect on the mood and attitude of the farmer and this makes biosecurity more difficult.

**Table 6:** Reported suggestions for improvement of on-farm biosecurity reported by professionals visiting farms in their work

**Suggestions for improved biosecurity**	**(n)**
Protective clothing made available on-farm; clean, warm and of adequate size (f)	65
Information to farmers, making farmers more aware, active dialogue with farmers, move the responsibility to the farmers (com)	58
Warm water, soap, wash basin and paper-towels available on-farm (f)	57
Hard surface and water hose with adequate pressure available on-farm to clean boots and equipment, adequately located (f)	34
Hygiene barriers, separating clean area from dirty area to avoid recontamination (f)	22
Education, both for farmers and professionals	22
Always keep up good routines; clean clothing and good hygiene (own)	19
Separate load-out areas, people present on the farm who can handle the animals when loading (f)	14
Bring disposable gloves, hand disinfectant etc. in the car (own)	14
Hand disinfectant (f)	11
Foot bath (f)	10
Protective clothing suitable in wintertime (low temperature) (w)	10
Consider risk for disease spread when planning routes between farms (own)	10
Hand washing (own)	10
National biosecurity guidelines for both farmers and professionals. All professionals should require the same level of conditions for biosecurity from the farmers (e.g. always requiring clean boots and protective clothing).	9
Clean surfaces available on-farm where to put the equipment (f)	8
Good general hygiene on-farm (f)	8
Farmers should be open about the current disease status of the farm (com)	8
Journal articles, information via organizations, brochures (com)	7
More protective clothes and washing machines provided by the employer (w)	7
Written routines (w)	7
A national on-farm biosecurity programme, with a certification worth more than just a paper	6
Active dialogue among colleagues (w)	6
Better routines in the car (w)	6
Professional attitude, serve as the good example, point out the advantages with biosecurity (com)	5
Other*	29

Based on an open ended question in a questionnaire to Swedish veterinarians, AI-technicians, animal welfare inspectors, livestock hauliers and hoof trimmers in 2012–2013.

(f) = on-farm, (w) = workplace, (com) = communication.

*Other = Suggestions for improved on-farm biosecurity for visitors reported by less than five responders, for example: Storage boxes for used protective clothing in the car, Equipment available on the farm, e.g. for hoof trimming, Sign on the door, stating no entrance without permission from the farmer, Already when planning for new stables, biosecurity and disease prevention should be included, Have higher demands , “have the guts to speak up to farmers and colleagues”, Provide financial incentives, Sell protective clothing and hand disinfectant to farmers, To clean equipment, Equipment that is easy to clean.

In addition to the open ended questions, many of the respondents had written comments to the other questions (not included in the tables). Many of the comments gave additional information to the answers given and some comments clarified specific problems faced by certain groups of visitors. For example, the inspectors wrote that they always brought their own clean protective clothing, i.e. there was seldom need either for farmers to request more from them or, for them to ask the farmer for better conditions. Inspectors were also very concerned not to spread disease, expressing worries that it would give the inspectors as a group a bad reputation if they did. There were also comments related to how their task affected their work, and that they could experience threatening situations that sometimes affected the possibility to keep up good biosecurity. For cattle farms, there were many comments emphasising the difference between dairy herds and beef herds, where the biosecurity was perceived as better in the dairy herds. Related to pig herds there were comments that they in general were better compared to other species, but also that the routines were better while the eradication programme against Aujeszky’s disease was still ongoing. There were also comments stating that farmers in general, and even horse owners, almost always provided soap, warm water and towels twenty years ago, while this was less common nowadays. Regarding the horse stables, there were numerous comments about non-existing biosecurity, horse owners questioning the need for hand wash or protective clothing as well as comments related to horses being scared of the ordinary coats. Several veterinarians reported not using protective coats when treating horses for this reason. The status of the protective clothing provided by farmers generated many comments. In addition to recurrent comments about wet, cold and dirty clothes there were also comments about boots containing rat’s nests, mouse droppings or spider webs.

## Discussion

Many of the respondents experienced obstacles for on-farm biosecurity, a remarkable number of the reported obstacles related to the very basics of biosecurity. Some respondents described a worsening situation. Sweden has historically had a strategy to control and eradicate diseases through specific programmes [29], and after eradication the routines are gradually relaxed, as was seen with Aujezsky’s disease (Sweden was declared officially free in 1996 [30]). Except for an outbreak of PRRS in 2007 and two vector borne diseases (Bluetongue and Schmallenberg), Sweden has not had any large outbreak of an exotic animal disease for decades [29-32], this may have had a negative impact on farmer awareness.

Responsibility and expectancy seem to be key issues mentioned by several respondents both in this study and in another current study focusing on the farmers’ perspectives (unpublished data). The visitors reported that the on-farm conditions did not allow an adequate level of biosecurity and that many farmers did not require any biosecurity routines, whereas farmers reported that they expect all visitors to be professional and take responsibility for not spreading any diseases. Recent national legislation in the area may help in clarifying the responsibilities [33] as well as the European Union proposal for a new Animal Health Law [7].

Several veterinarians reported that they had repeatedly asked farmers for improvements, but some concluded that veterinarians as a group have not been explicit enough. The benefits of veterinarians communicating messages about biosecurity to farmers, as well as farmers’ preference for receiving biosecurity information from their veterinarian has been identified in other studies [8,18,19,34-36].

There were differences in the perceived conditions among different categories of visitors for the same category of farm. In part this can be explained by different types of farms visited, e.g. animal welfare inspectors visit another group of cattle farmers compared to AI-technicians. But there were also differences related to the task performed on the farm. For example, the animal welfare inspectors do not visit the farm at the farmer’s request, but more likely the opposite. They found it difficult to demand anything at all, and were afraid to be blamed for any disease introduction. Another example was the livestock hauliers who suggested that hand hygiene facilities be provided where they enter the farm, not only at the entrance for other visitors. These aspects should be kept in mind when developing biosecurity recommendations for different categories of professionals.

Limited access to water and soap was a general problem. This is surprising since the need for hand hygiene is old knowledge, described by Semmelweis in 1847 [37]. There are situations, e.g. on pasture, with no access to running water, but this can easily be solved, as was suggested, by bringing a water container, soap and a clean bucket. Several of the participants in the study also reported efforts to keep their hands clean despite poor conditions on the farm, e.g. stopping at a gas-station to wash their hands or using hand disinfectants and wipes in the car. Not all visitors may make this extra effort, and the effects of hand disinfectants without prior washing may not be sufficient [38]. In the extensive work to improve hand hygiene in human health, accessibility has been identified as an important factor for compliance with hand washing and disinfection routines [37].

Some responses indicated a lack of understanding among farmers of how infectious disease agents can spread through indirect contact. There were numerous comments on how protective clothing, when provided, was cold, damp and dirty. Clothes and boots in poor condition will not be used and will not fulfil the purpose of avoiding contamination of the visitor. There is a problem if farmers believe clothes and boots in such poor conditions to be adequate, but this may not only be related to lack of understanding. There are other influencing factors, like personality traits, which are discussed in a Canadian study where farmers were observed taking biosecurity risks through reusing shoe protections from the garbage [21].

The poor biosecurity conditions reported for farms with horses are alarming and there is obviously room for improvement. Need for improvement of biosecurity among horse owners and horse practitioners has also been identified in other countries [3,39,40]

Salmonella was the disease that most respondents were afraid to spread between farms. This may be related to the farm restrictions in the Swedish salmonella control programme [41]. The other diseases mentioned reflected the endemic disease situation in Sweden. However, some responses only included exotic animal diseases regulated by law. This may indicate an awareness of these diseases, but may also reflect an unawareness of the actual situation in Sweden. An increasing number of veterinarians in Sweden have a veterinary degree from abroad [42], from countries where these diseases may be endemic.

Several studies have concluded that people working with livestock are at higher risk of contracting zoonotic diseases and for colonisation with MRSA [28,43]. In our study, 44% mentioned one or more zoonotic agents they were afraid to contract while working. From an Australian study it was reported that 35.3% of the veterinarians were concerned or very concerned for either themselves or for colleagues [27], but since the questions were asked in different ways these figures are not directly comparable. Ringworm was the disease that most persons mentioned as one of the diseases they were afraid to contract in their daily work, the same result was found in a study among veterinarians in the US [26]. Ringworm was also reported to be the most common zoonosis contracted by veterinarians both in Oregon and in South Africa [25,44] and is probably a highly relevant zoonosis in Sweden as well since it is endemic in livestock. Salmonella came in second place, although the prevalence of salmonella in Swedish livestock is quite low [41]. This high ranking could be due to absence of other severe zoonotic infections such as brucellosis and tuberculosis [29,30]. Multi-resistant bacteria and MRSA came in third place, although the prevalence is still believed to be low in Sweden [45]. There were also six persons stating that they were afraid to contract rabies, even though rabies was last confirmed in a Swedish animal in 1886 [30]. In recent years, however, there has been an increased illegal import of dogs to Sweden [46] and the fear of contracting rabies may be related to this.

This study used a convenience sample. Due to the distribution methods used, overall response rates cannot be assessed. How non-responders might differ from the responders, and the representativeness of the results, cannot be assessed either. It is possible that people with a particular interest in biosecurity, and perhaps frustrated by the lack of it, were more prone to answer the questionnaire, leading to a bias towards reporting poor biosecurity. Although persons with an interest in biosecurity may be overrepresented among the respondents, obstacles reported are likely to be generally present. However, the factors motivating biosecurity could be different among the non-respondents compared to the respondents. Despite these limitations, this study has captured views and opinions regarding on-farm biosecurity from groups of professionals where previously no information was available.

The many different (largely unknown) underlying factors affecting the types of farms visited and the uncertainty in the representativeness of both respondents and farms they visited was the reason for keeping to descriptive statistics, more advanced statistical methods would probably not be more informative.

There are a number of factors affecting biosecurity, both from the farmers’ and from the visitors’ perspectives. Lack of knowledge can be one reason for not implementing biosecurity routines [16]. However, human behaviour is complex and it is well known that not only knowledge is needed to change behaviour [47]. It is important also to understand the experienced obstacles and motivators for biosecurity. This study is part of a project in which farmers are also asked about hindrances and motivators for biosecurity. Trying to approach the on-farm biosecurity related to professionals visiting farms from two different perspectives will add to the understanding of this issue.

## Conclusions

Many of the respondents reported obstacles for keeping adequate biosecurity related to on-farm conditions, and a large proportion of farms visited were reported to lack the very basics for visitors’ biosecurity. Different visitors seemed to have different conditions for maintaining biosecurity on farms. There was a gap when it came to responsibility and this need to be clarified; visitors need to take responsibility for avoiding spread of disease, while farmers need to assume responsibility for providing adequate conditions for on-farm biosecurity.

## Competing interests

There were no competing interests.

## Authors’ contributions

MN and SS designed the study. MN developed the questionnaire, collected data, analysed the data and drafted the manuscript. SSL participated in the analyses of data and revised the manuscript. Both authors read and approved the final manuscript.

## Supplementary Material

Additional file 1 **Questionnaire, translated to English.** Questionnaire regarding biosecurity to persons visiting farms in their profession, translated to English. Click here for file

Additional file 2 **Questionnaire, Swedish.** Questionnaire regarding biosecurity to persons visiting farms in their profession, Swedish original version. Click here for file
