# Supplementary material for: On-farm biosecurity as perceived by professionals visiting Swedish farms
Source: Acta Vet Scand. 2014 May 9;56(1):28. doi: 10.1186/1751-0147-56-28 (PMC4036743; doi:10.1186/1751-0147-56-28)
Supplement: Additional file 1 — Questionnaire, translated to English. Questionnaire regarding biosecurity to persons visiting farms in their profession, translated to English. [file 1751-0147-56-28-S1.pdf]

**NB! This is a rough translation of the Swedish text, and may not exactly reflect the Swedish phrasing.**

**Please state your profession**

- ❑ Veterinarian
- ❑ AI-technician
- ❑ Livestock haulier
- ❑ Advisor
- ❑ Inspector
- ❑ Other

If other, please specify: ___________________________________

**Please state your age**

Tick your age category (in years):

- ❑ >20
- ❑ 21-35
- ❑ 36-50
- ❑ 51-65
- ❑ >66

**What type of farms with animals do you visit in your work?**

Enter one or more options

- ❑ Cattle holdings
- ❑ Pig holdings
- ❑ Sheep and goat holdings
- ❑ Horse stables
- ❑ I never visit any farms with animals in my profession

**Approximately how many farms with animals do you visit per week?**

- ❑ Less than one
- ❑ 1-10
- ❑ 11-20
- ❑ More than 20

**If you visit farms with different species of animals, do you perceive any differences in biosecurity routines between owners of different animal species?**

- ❑ Yes
- ❑ No
- ❑ Do not know/decline to answer

**If you do not visit farms with cattle, please continue to the next page**

**1) When you visit CATTLE farms, what proportion of the farms tend to have the following:**

| | none | almost none | less than half | approx. half | more than half | almost all | all | decline reply |
|---|---|---|---|---|---|---|---|---|
| **Hygiene barrier**, i.e. a place where it is clear to you as a visitor that you should change boots and outer clothing | ❑ | ❑ | ❑ | ❑ | ❑ | ❑ | ❑ | ❑ |
| **Protective clothing** for visitors | ❑ | ❑ | ❑ | ❑ | ❑ | ❑ | ❑ | ❑ |
| **Boots** for visitors | ❑ | ❑ | ❑ | ❑ | ❑ | ❑ | ❑ | ❑ |
| Possibilities for **hand washing** | ❑ | ❑ | ❑ | ❑ | ❑ | ❑ | ❑ | ❑ |
| Possibilities for **hand disinfection,** e.g. alcohol based hand disinfectants | ❑ | ❑ | ❑ | ❑ | ❑ | ❑ | ❑ | ❑ |

Comments, optional **:**

**2) When you are entering the animal stables on farms with CATTLE**

| | none | almost none | less than half | approx. half | more than half | almost all | all | decline reply |
|---|---|---|---|---|---|---|---|---|
| Do you experience that the animal owners require you to wear protective clothing? | ❑ | ❑ | ❑ | ❑ | ❑ | ❑ | ❑ | ❑ |

Comments, optional:

**If you do not visit farms with pigs, please continue to the next page**

**3) When you visit PIG farms, what proportion of the farms tend to have the following:**

| | none | almost none | less than half | approx. half | more than half | almost all | all | decline reply |
|---|---|---|---|---|---|---|---|---|
| **Hygiene barrier**, i.e. a place where it is clear to you as a visitor that you should change boots and outer clothing | ❑ | ❑ | ❑ | ❑ | ❑ | ❑ | ❑ | ❑ |
| **Protective clothing** for visitors | ❑ | ❑ | ❑ | ❑ | ❑ | ❑ | ❑ | ❑ |
| **Boots** for visitors | ❑ | ❑ | ❑ | ❑ | ❑ | ❑ | ❑ | ❑ |
| Possibilities for **hand washing** | ❑ | ❑ | ❑ | ❑ | ❑ | ❑ | ❑ | ❑ |
| Possibilities for **hand disinfection,** e.g. alcohol based hand disinfectants | ❑ | ❑ | ❑ | ❑ | ❑ | ❑ | ❑ | ❑ |

Comments, optional**:**

**4) When you are entering the animal stables on farms with PIGS**

| | none | almost none | less than half | approx. half | more than half | almost all | all | decline reply |
|---|---|---|---|---|---|---|---|---|
| Do you experience that animal owners require you to wear protective clothing? | ❑ | ❑ | ❑ | ❑ | ❑ | ❑ | ❑ | ❑ |

Comments, optional:

**If you do not visit farms with sheep or goats, please continue to the next page**

---

**5) When you visit SHEEP or GOAT farms, what proportion of the farms tend to have the following:**

| | none | almost none | less than half | approx. half | more than half | almost all | all | decline reply |
|---|---|---|---|---|---|---|---|---|
| **Hygiene barrier**, i.e. a place where it is clear to you as a visitor that you should change boots and outer clothing | ❑ | ❑ | ❑ | ❑ | ❑ | ❑ | ❑ | ❑ |
| **Protective clothing** for visitors | ❑ | ❑ | ❑ | ❑ | ❑ | ❑ | ❑ | ❑ |
| **Boots** for visitors | ❑ | ❑ | ❑ | ❑ | ❑ | ❑ | ❑ | ❑ |
| Possibilities for **hand washing** | ❑ | ❑ | ❑ | ❑ | ❑ | ❑ | ❑ | ❑ |
| Possibilities for **hand disinfection,** e.g. alcohol based hand disinfectants | ❑ | ❑ | ❑ | ❑ | ❑ | ❑ | ❑ | ❑ |

Comments, optional**:**

**6) When you are entering the animal stables on farms with SHEEP or GOATS**

| | none | almost none | less than half | approx. half | more than half | almost all | all | decline reply |
|---|---|---|---|---|---|---|---|---|
| Do you experience that animal owners require you to wear protective clothing? | ❑ | ❑ | ❑ | ❑ | ❑ | ❑ | ❑ | ❑ |

Comments, optional:

**If you do not visit farms with horses, please continue to the next page**

**7) When you visit HORSE farms, what proportion of the farms tend to have the following:**

| | none | almost none | less than half | approx. half | more than half | almost all | all | decline reply |
|---|---|---|---|---|---|---|---|---|
| **Hygiene barrier**, i.e. a place where it is clear to you as a visitor that you should change boots and outer clothing | ❑ | ❑ | ❑ | ❑ | ❑ | ❑ | ❑ | ❑ |
| **Protective clothing** for visitors | ❑ | ❑ | ❑ | ❑ | ❑ | ❑ | ❑ | ❑ |
| **Boots** for visitors | ❑ | ❑ | ❑ | ❑ | ❑ | ❑ | ❑ | ❑ |
| Possibilities for **hand washing** | ❑ | ❑ | ❑ | ❑ | ❑ | ❑ | ❑ | ❑ |
| Possibilities for **hand disinfection,** e.g. alcohol based hand disinfectants | ❑ | ❑ | ❑ | ❑ | ❑ | ❑ | ❑ | ❑ |

Comments, optional**:**

**8) When you are entering the animal stables on farms with HORSES**

| | none | almost none | less than half | approx. half | more than half | almost all | all | decline reply |
|---|---|---|---|---|---|---|---|---|
| Do you experience that animal owners require you to wear protective clothing? | ❑ | ❑ | ❑ | ❑ | ❑ | ❑ | ❑ | ❑ |

Comments, optional**:**

**9) If you are a livestock haulier, in what proportion of farms where you deliver or collect animals do you need to enter the animal stables?**

| | none | almost none | less than half | approx. half | more than half | almost all | all | never visit this type |
|---|---|---|---|---|---|---|---|---|
| Pig farms | ❑ | ❑ | ❑ | ❑ | ❑ | ❑ | ❑ | ❑ |
| Cattle farms | ❑ | ❑ | ❑ | ❑ | ❑ | ❑ | ❑ | ❑ |
| Sheep- and goat farms | ❑ | ❑ | ❑ | ❑ | ❑ | ❑ | ❑ | ❑ |

Comments, optional**:**

**10) When you visit a farm, are your own biosecourity routines:**

❑ Always the same

❑ Different at different farms

Comments, optional **:**

**11) How important are the following factors for your own biosecurity routines when you visit a farm?**

| | un-important | less important | quite important | very important |
|---|---|---|---|---|
| **Animal species** present on the farm | ❑ | ❑ | ❑ | ❑ |
| Herd **size** | ❑ | ❑ | ❑ | ❑ |
| If the farm provides **protective clothing** | ❑ | ❑ | ❑ | ❑ |
| **Requirements from farmer** | ❑ | ❑ | ❑ | ❑ |
| Requirements within my **own organization** | ❑ | ❑ | ❑ | ❑ |
| It is a competitive advantage to be perceived as being well aware of hygiene | ❑ | ❑ | ❑ | ❑ |
| Own **wish not to spread disease between farms** | ❑ | ❑ | ❑ | ❑ |
| Current **outbreaks** or **eradication programmes** | ❑ | ❑ | ❑ | ❑ |
| Other | ❑ | ❑ | ❑ | ❑ |

If other, please indicate:

______________________________

______________________________

______________________________

Comments, optional**:**

**12) Have you as a visitor asked farmers to provides means to make it possible for you to keep an adequate level of biosecurity on their farm?**
(One example could be if you asked the farmer to provide boots or hand disinfectant)

- ❑ Never
- ❑ A few times
- ❑ Many times

Comments, optional**:**

**13) What routines do persons within YOUR PROFESSION generally use when visiting farms, and either being in direct contact with animals or just entering the animal stables?**
Indicate what proportion of the visits you believe the following routines are applied:

| | none | almost none | less than half | approx. half | more than half | almost all | all | don’t know |
|---|---|---|---|---|---|---|---|---|
| Use of **protective clothing provided by the farm** or a new **clean change** in each farm | ❑ | ❑ | ❑ | ❑ | ❑ | ❑ | ❑ | ❑ |
| Use of **boots provided by the farm** or **shoe covers** (single use) or **clean boots** between each farm | ❑ | ❑ | ❑ | ❑ | ❑ | ❑ | ❑ | ❑ |
| **Washing** hands before entering the stable or before animal contact | ❑ | ❑ | ❑ | ❑ | ❑ | ❑ | ❑ | ❑ |
| **Disinfecting hands** (e.g. alcohol based hand disinfectant) **before entering a stable or before animal contact** | ❑ | ❑ | ❑ | ❑ | ❑ | ❑ | ❑ | ❑ |
| **Clean equipment** between each farm | ❑ | ❑ | ❑ | ❑ | ❑ | ❑ | ❑ | ❑ |

Comments, optional**:**

**14) What routines do YOU generally apply visiting farms, and either being in direct contact with animals or entering the animal stables?**

Indicate what proportion of the visits you apply the following routines:

| | none | almost none | less than half | approx. half | more than half | almost all | all | never enter stables | decline reply |
|---|---|---|---|---|---|---|---|---|---|
| Use of **protective clothing provided by the farm** or a new **clean change** in each farm | ❑ | ❑ | ❑ | ❑ | ❑ | ❑ | ❑ | ❑ | ❑ |
| Use of **boots provided by the farm** or **shoe protections** (single use) or **clean boots** between each farm | ❑ | ❑ | ❑ | ❑ | ❑ | ❑ | ❑ | ❑ | ❑ |
| **Washing** hands before entering the stable or before animal contact | ❑ | ❑ | ❑ | ❑ | ❑ | ❑ | ❑ | ❑ | ❑ |
| **Disinfecting hands** (e.g. alcohol based hand disinfectant) **before entering a stable or animal contact** | ❑ | ❑ | ❑ | ❑ | ❑ | ❑ | ❑ | ❑ | ❑ |
| **Clean equipment** between each farm | ❑ | ❑ | ❑ | ❑ | ❑ | ❑ | ❑ | ❑ | ❑ |

Comments, optional**:**

**15) Are there any specific infectious agents that you are afraid to spread between farms through your work?**

❑ Yes

❑ No

If yes, please indicate which (one or more):

_______________________________________________

_______________________________________________

_______________________________________________

_______________________________________________

_______________________________________________

_______________________________________________

_______________________________________________

**16) Are there any specific infectious agents that you are afraid to contract yourself through your work??**

- ❑ Yes
- ❑ No

If yes, please indicate which (one or more):

________________________________________________________________
________________________________________________________________
________________________________________________________________
________________________________________________________________
________________________________________________________________
________________________________________________________________
________________________________________________________________
________________________________________________________________

**17) What do you think about your own biosecurity routines in association to farm visits?**

My own routines are:

- ❑ Very good
- ❑ Sufficient
- ❑ Insufficient:
- ❑ It varies between different farms

Comments, optional:

**18) Do you perceive any obstacles that prevent you from keeping up an adequate level of biosecurity while working?**

- ❑ Yes
- ❑ No

If yes, please indicate what you experience as the most important hinders:

________________________________________________________________
________________________________________________________________
________________________________________________________________
________________________________________________________________
________________________________________________________________
________________________________________________________________
________________________________________________________________
________________________________________________________________
________________________________________________________________
________________________________________________________________
________________________________________________________________
________________________________________________________________

**19) What do you consider to be important factors for being able to improve on farm biosecurity and biosecurity within your profession?**

Please mention practical examples as well as factors that you believe could motivate better biosecurity

- - - - - - - - - - - - - - - - - - - - - - - - - - - - - - - - - - - - - - - -

**Do you want to take part of the results when they are ready? (If you want to you can cut off this part and send it in a separate envelope or email the information directly to maria.noremark@sva.se)**

- ❑ No thank you.
- ❑ Yes please, through e-mail (indicate address below)
- ❑ Yes please, through ordinary mail (indicate name and postal address )

If yes, indicate postal address or e-mail

**THANK you for your participation!**
