# Supplementary material for: On-farm biosecurity as perceived by professionals visiting Swedish farms
Source: Acta Vet Scand. 2014 May 9;56(1):28. doi: 10.1186/1751-0147-56-28 (PMC4036743; doi:10.1186/1751-0147-56-28)
Supplement: Additional file 2 — Questionnaire, Swedish. Questionnaire regarding biosecurity to persons visiting farms in their profession, Swedish original version. [file 1751-0147-56-28-S2.pdf]

Ange den yrkeskategori du tillhör

- ❑ Veterinär
- ❑ Husdjurstekniker
- ❑ Djurtransportör
- ❑ Rådgivare
- ❑ Inspektör
- ❑ Annan

Om annan, ange vilken: ___________________________________

Hur gammal är du?

Ange vilket åldersintervall (siffror i år) du befinner dig i:

- ❑ >20
- ❑ 21-35
- ❑ 36-50
- ❑ 51-65
- ❑ >66

Vilken typ av gårdar med djur besöker du i ditt yrke?

Ange ett eller flera alternativ

- ❑ Nötbesättningar
- ❑ Grisbesättningar
- ❑ Får- och getbesättningar
- ❑ Hästanläggningar
- ❑ Är aldrig ute på några gårdar med djur i mitt yrke

Ungefär hur många gårdar med djur besöker du per vecka?

- ❑ Färre än 1
- ❑ 1-10
- ❑ 11-20
- ❑ Fler än 20

Om du besöker gårdar med olika djurslag, upplever du att det är skillnader i smittskyddsrutiner mellan djurägare med olika djurslag?

- ❑ Ja
- ❑ Nej
- ❑ Vet ej/avstår svar

Om du inte besöker besättningar med nötkreatur forstätt på nästa sida.

---

1) När du besöker besättningar med NÖTKREATUR, i hur stor andel av besättningarna brukar följande finnas:

	inga	nästan inga	färre än hälften	ungefär hälften	fler är hälften	nästan alla	alla	avstår svar
**Hygiengräns**, dvs. plats där det är tydligt att du som besökare ska byta skor och kläder	❑	❑	❑	❑	❑	❑	❑	❑
**Skyddskläder** för besökare	❑	❑	❑	❑	❑	❑	❑	❑
**Stövlar** för besökare	❑	❑	❑	❑	❑	❑	❑	❑
Möjligheter till **handtvätt**	❑	❑	❑	❑	❑	❑	❑	❑
Möjlighet till **handdesinfektion**, t.ex. handsprit	❑	❑	❑	❑	❑	❑	❑	❑

Kommentarer, frivilligt:

2) När du ska gå in i djurstallarna i besättningar med NÖTKREATUR

	inga	nästan inga	färre än hälften	ungefär hälften	fler är hälften	nästan alla	alla	avstår svar
Upplever du att djurägarna har krav på att du som besökare ska använda skyddskläder?	❑	❑	❑	❑	❑	❑	❑	❑

Kommentarer, frivilligt:

Om du inte besöker besättningar med GRISAR forstätt på nästa sida.

3) När du besöker besättningar med GRISAR, i hur stor andel av besättningarna brukar följande finnas:

	inga	nästan inga	färre än hälften	ungefär hälften	fler är hälften	nästan alla	alla	avstår svar
**Hygiengräns**, dvs. plats där det är tydligt att du som besökare ska byta skor och kläder	❑	❑	❑	❑	❑	❑	❑	❑
**Skyddskläder** för besökare	❑	❑	❑	❑	❑	❑	❑	❑
**Stövlar** för besökare	❑	❑	❑	❑	❑	❑	❑	❑
Möjligheter till **handtvätt**	❑	❑	❑	❑	❑	❑	❑	❑
Möjlighet till **handdesinfektion**, t.ex. handsprit	❑	❑	❑	❑	❑	❑	❑	❑

Kommentarer, frivilligt:

4) När du ska gå in i djurstallarna i besättningar med GRISAR

	inga	nästan inga	färre än hälften	ungefär hälften	fler är hälften	nästan alla	alla	avstår svar
Upplever du att djurägarna har krav på att du som besökare ska använda skyddskläder?	❑	❑	❑	❑	❑	❑	❑	❑

Kommentarer, frivilligt:

Om du inte besöker besättningar med får eller getter forstätt på nästa sida.

---

5) När du besöker besättningar med FÅR eller GETTER, i hur stor andel av besättningarna brukar följande finnas:

	inga	nästan inga	färre än hälften	ungefär hälften	fler är hälften	nästan alla	alla	avstår svar
**Hygiengräns**, dvs. plats där det är tydligt att du som besökare ska byta skor och kläder	❑	❑	❑	❑	❑	❑	❑	❑
**Skyddskläder** för besökare	❑	❑	❑	❑	❑	❑	❑	❑
**Stövlar** för besökare	❑	❑	❑	❑	❑	❑	❑	❑
Möjligheter till **handtvätt**	❑	❑	❑	❑	❑	❑	❑	❑
Möjlighet till **handdesinfektion**, t.ex. handsprit	❑	❑	❑	❑	❑	❑	❑	❑

Kommentarer, frivilligt:

6) När du ska gå in i djurstallarna i besättningar med FÅR eller GETTER

	inga	nästan inga	färre än hälften	ungefär hälften	fler är hälften	nästan alla	alla	avstår svar
Upplever du att djurägarna har krav på att du som besökare ska använda skyddskläder?	❑	❑	❑	❑	❑	❑	❑	❑

Kommentarer, frivilligt:

Om du inte besöker anläggningar med hästar forstätt på nästa sida.

7) När du besöker anläggningar med HÄSTAR, i hur stor andel av besättningarna brukar följande finnas:

	inga	nästan inga	färre än hälften	ungefär hälften	fler är hälften	nästan alla	alla	avstår svar
**Hygiengräns**, dvs. plats där det är tydligt att du som besökare ska byta skor och kläder	❑	❑	❑	❑	❑	❑	❑	❑
**Skyddskläder** för besökare	❑	❑	❑	❑	❑	❑	❑	❑
**Stövlar** för besökare	❑	❑	❑	❑	❑	❑	❑	❑
Möjligheter till **handtvätt**	❑	❑	❑	❑	❑	❑	❑	❑
Möjlighet till **handdesinfektion**, t.ex. handsprit	❑	❑	❑	❑	❑	❑	❑	❑

Kommentarer, frivilligt:

8) När du ska gå in i djurstallarna i anläggningar med HÄSTAR

	inga	nästan inga	färre än hälften	ungefär hälften	fler är hälften	nästan alla	alla	avstår svar
Upplever du att djurägarna har krav på att du som besökare ska använda skyddskläder?	❑	❑	❑	❑	❑	❑	❑	❑

Kommentarer, frivilligt:

9) **Om du är transportör, i de besättningar där du lämnar eller hämtar djur, i hur stor andel måste du gå in i djurstallarna?**

	inga	nästan inga	färre än hälften	ungefär hälften	fler är hälften	nästan alla	alla	är aldrig i sådana besättn ingar
I grisbesättningar	❑	❑	❑	❑	❑	❑	❑	❑
I nötbesättningar	❑	❑	❑	❑	❑	❑	❑	❑
I får- och getbesättningar	❑	❑	❑	❑	❑	❑	❑	❑

Kommentarer, frivilligt:

10) **När du besöker en besättning, är dina egna smittskyddsrutiner**

❑ Alltid samma

❑ Olika i olika besättningar

Kommentar, frivilligt:

11) **Hur viktiga är följande faktorer för dina egna smittskyddsrutiner när du besöker en gård?**

	oviktigt	mindre viktigt	ganska viktigt	väldigt viktigt
**Djurslag** på gården	❑	❑	❑	❑
Besättnings**storlek**	❑	❑	❑	❑
**Tillgång till skyddskläder** på gården	❑	❑	❑	❑
**Djurägarens krav**	❑	❑	❑	❑
Den **egna organisationens** krav	❑	❑	❑	❑
Att det är en **konkurrensfördel** att uppfattas som hygienmedveten	❑	❑	❑	❑
Egen **önskan om att inte sprida smitta** mellan gårdar	❑	❑	❑	❑
Pågående **utbrott** eller **bekämpningsprogram**	❑	❑	❑	❑
Annat	❑	❑	❑	❑

Annat, ange vad:

Kommentarer, frivilligt:

12) Har du som besökare bett djurägare att de ska skapa förutsättningar för att du ska kunna ha ett bra smittskydd?

(Ett exempel kan vara om du ställt krav på att det ska finnas stövlar till dig som besökare eller att det ska finnas handsprit)

- ❑ Aldrig
- ❑ Någon gång
- ❑ Många gånger

Kommentar, frivilligt:

13) Vilka rutiner brukar DIN YRKESKATEGORI generellt använda vid gårdsbesök då de är inne i stallar eller i direktkontakt med djur?

Ange i hur stor andel av besöken du tror att följande rutiner används.

	inga	nästan inga	färre än hälften	ungefär hälften	fler är hälften	nästan alla	alla	vet ej
Använder **gårdens skyddskläder** eller **rent ombyte** i varje besättning	❑	❑	❑	❑	❑	❑	❑	❑
Använder **gårdens stövlar** eller **skoskydd** (engångs), eller **rengör egna stövlar** mellan varje gård	❑	❑	❑	❑	❑	❑	❑	❑
**Tvättar händer** före inträde i stall eller kontakt med djuren	❑	❑	❑	❑	❑	❑	❑	❑
Desinfekterar händer (t.ex. **handsprit**) **före inträde** i stall eller kontakt med djuren	❑	❑	❑	❑	❑	❑	❑	❑
**Rengör utrustning** mellan varje gård	❑	❑	❑	❑	❑	❑	❑	❑

Kommentar, frivilligt:

14) Vilka rutiner brukar DU generellt använda vid gårdsbesök då du är inne i stallar eller i direktkontakt med djur?

Ange i hur stor andel av besöken du använder följande rutiner.

	inga	nästan inga	färre än hälften	ungefär hälften	fler än hälften	nästan alla	alla	är aldrig inne i stallar	avstår svar
Använder gårdens skyddskläder eller rent ombyte i varje besättning	❑	❑	❑	❑	❑	❑	❑	❑	❑
Använder gårdens stövlar eller skoskydd (engångs), eller rengör egna stövlar mellan varje gård	❑	❑	❑	❑	❑	❑	❑	❑	❑
Tvättar händer före inträde i stall eller kontakt med djuren	❑	❑	❑	❑	❑	❑	❑	❑	❑
Desinfekterar händer (t.ex. handsprit) före inträde i stall eller kontakt med djuren	❑	❑	❑	❑	❑	❑	❑	❑	❑
Rengör utrustning mellan varje gård	❑	❑	❑	❑	❑	❑	❑	❑	❑

Kommentar, frivilligt:

15) Finns det något eller några särskilda smittämnen som du är rädd för att sprida mellan besättningar genom din yrkesutövning?

❑ Ja

❑ Nej

Om ja, ange gärna vilket eller vilka:

____

____

____

____

____

____

____

16) Finns det något eller några smittämnen som du är rädd för att själv drabbas av i din yrkesutövning?

- ❑ Ja
- ❑ Nej

Om ja, ange gärna vilket eller vilka

____
____
____
____
____
____
____
____

17) Vad tycker du om dina egna smittskyddsrutiner i samband med gårdsbesök?

Mina rutiner är:

- ❑ Mycket bra
- ❑ Tillräckliga
- ❑ Otillräckliga
- ❑ Det varierar mellan olika gårdar

Kommentar, frivilligt:

18) Upplever du att det finns något som hindrar dig från att ha ett bra smittskydd i din yrkesutövning?

- ❑ Ja
- ❑ Nej

Om ja, ange i så fall det som du upplever som de viktigaste hindren:

____
____
____
____
____
____
____
____
____
____
____
____

19) Vad tycker du själv är viktiga faktorer för att kunna förbättra smittskyddet både på gårdsnivå och inom din yrkeskategori?

Ta gärna upp både praktiska exempel och faktorer som du tycker kan motivera till ett bättre smittskydd.

____
____
____
____
____
____
____
____
____
____
____
____
____
____
____
____
____
____
____

- -

20) Vill du ta del av analysresultaten när de är klara? (om du vill kan du klippa av denna del av enkäten och skicka separat eller mejla uppgifter om att du vill ta del av enkäten till maria.noremark@sva.se)

- ❑ Nej tack.
- ❑ Ja tack, via mejl (ange mejladress nedan)
- ❑ Ja tack, via brev (ange namn och postadress nedan)

Om ja, ange mejladress eller namn och postadress:

____
____
____
____

TACK för din medverkan!

Skicka enkäten i bifogat kuvert till:
SVA
ESS, Maria Nöremark
751 89 Uppsala
